# *Drosophila* view on glia in peripheral sensory neuropathy

**DOI:** 10.4103/NRR.NRR-D-25-00682

**Published:** 2025-09-29

**Authors:** Steffen Kautzmann, Christian Klämbt

**Affiliations:** University of Münster, Institut für Neuro- und Verhaltensbiologie, Münster, Germany

Peripheral sensory neurons perceive external signals and convey signals to the central nervous system (CNS). Information transmission occurs via often extremely long axons and timely reactions of the animal require a fast conductance velocity. This not only depends on axonal diameter and insulation by glial processes, but it requires the structural integrity of the axon. Progressive degradation of axons is a common pathophysiological event that, for example, can be triggered by high glucose levels as seen in patients with diabetes mellitus or caused by several chemotherapeutic agents.

Diabetic peripheral neuropathy is a major complication in diabetes mellitus affecting roughly 30% of all patients, causing chronic pain and sensory loss. Similar to what is observed in chemotherapy-induced peripheral neuropathy, diabetic peripheral neuropathy primarily affects thin sensory axons. Motor axons appear to resist degeneration even upon partial demyelination. Despite the large number of patients affected worldwide, the molecular mechanisms underlying such peripheral neuropathies are not fully understood, preventing the development of effective therapeutic intervention strategies (Feldman et al., 2017; Suzuki et al., 2023). A surprising avenue towards deciphering mechanisms and unraveling therapeutic intervention targets is emerging from studies using the model organism Drosophila.

For example, work in Drosophila helped to elucidate axon intrinsic mechanisms underlying Wallerian degeneration. Key players in this process were identified, including the following pro-degenerative factors: the NAD(+) glycohydrolase encoded by *Sterile alpha and Armadillo motif* (*Sarm*), the ubiquitin E3 ligase encoded by *highwire* (hiw) and the mitogen-activated protein kinase kinase kinase encoded by *wallenda* (*wnd*), as well as the axon protective genes *Wnk kinase* (*Wnk*) and the NAD-producing enzyme Nicotinamide mononucleotide adenylyltransferase (Nmnat) (Bhattacharya, 2023).

In addition, extrinsic mechanisms were shown to be effective in controlling axonal degeneration. In diabetic peripheral neuropathy, high levels of glucose cause axonal degeneration. The development of hyperglycemia can be modeled in Drosophila, where it, as in human patients, leads to insulin resistance, obesity, heart and nephrocyte dysfunction (Kim et al., 2021). In addition, hyperglycemia also leads to glial dysfunction, which in turn results in sensory dysfunction (Suzuki et al., 2023). Quite similarly, microtubule disrupting agents that are frequently used in chemotherapeutic cocktails such as paclitaxel (Taxol) clearly act on Drosophila axon wrapping glial cells and also cause sensory axon degeneration (Bhattacharya et al., 2012). These findings place glial cells at the center of the understanding of diabetic peripheral neuropathy and chemotherapy-induced peripheral neuropathy.

**Figure 1:**
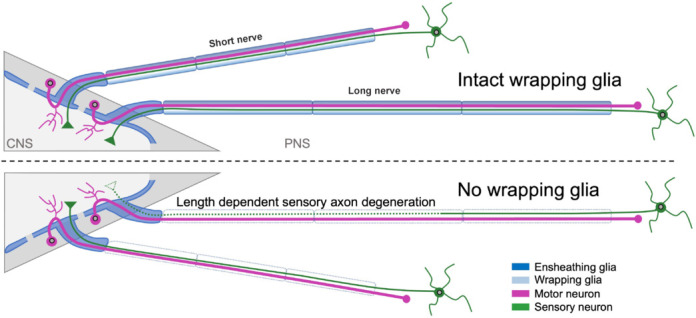
Length-dependent sensory axon degeneration in the peripheral nervous system (PNS) of *Drosophila*. The nervous system harbors sensory and motor neurons. They project their axons through segmental nerves in the PNS. The nerves innervating the thoracic or anterior abdominal segments are short, whereas the nerve running to the abdominal most segments span the entire animal and reach up to 3 mm in length. Cell bodies of the unipolar glutamatergic motor neurons (magenta) reside in the central nervous system (CNS) cortex. They project their dendrites into the neuropil (light grey) and their axon towards the peripheral musculature. The cell bodies of the cholinergic sensory neurons reside in the periphery. They synapse on targets in the neuropil. Importantly, the first part of the motor axon and the terminal part of all sensory axons are enwrapped by a special CNS-derived ensheathing glia (dark blue), while the majority of the axon in the PNS is wrapped by PNS-derived wrapping glia (light blue). Upon ablation of the peripheral wrapping glia sensory axons in these long nerves degenerate from the synaptic side, while their counterparts in the short nerves are not affected. In contrast, motor axons are not affected by glia ablation, even in the long nerves.

Three main glial cell types exist in vertebrates, of which two focus on axon wrapping. Oligodendrocytes in the CNS and Schwann cells in the peripheral nervous system (PNS). Interestingly, similar cells are also found in *Drosophila melanogaster* (**Figure 1**). In the larval PNS, axon wrapping glia differentiates in a Remak-type manner around sensory and motor axons. At the nerve root at the CNS/PNS boundary, the CNS-derived ensheathing glia wraps axons in the same fashion (**Figure 1**). Interestingly, in adult flies, these glial cells can form myelin-like structures (Rey et al., 2023).

Axon-specific labeling techniques, as well as electrophysiological measurements, demonstrated that sensory axons are smaller in diameter and have reduced conduction velocity compared to motor axons (Kottmeier et al., 2020; Kautzmann et al., 2025). Interestingly, sensory and motor axons show distinct interactions with the wrapping glia. Early on during development, sensory and motor axons are found in few large fascicles with no intermingling wrapping glial processes. During larval development, the wrapping glia preferentially separates motor axons, while sensory axons generally remain in larger fascicles. This could indicate that motor axons can grow to a larger diameter due to an intensified neuron-glia interface. In stark contrast to this, upon loss of peripheral wrapping glia, motor axons are found in normal numbers with an unchanged or even larger radial diameter. Sensory axons, however, show a length-dependent degeneration (Kautzmann et al., 2025; **Figure 1**, lower panel).

This length-dependent degeneration of specifically sensory neurons is reminiscent to what is found in many diabetes mellitus patients. They develop a peripheral neuropathy that is characterized by an early loss of unmyelinated sensory axons, and only a later degeneration of larger myelinated axons (Gonçalves et al., 2017). A recent work indicates that the primary trigger in diabetic neuropathy might be hyperglycemia-induced death of Schwann cells (Wu et al., 2024). Axonal degeneration and myelin abnormalities causing neuropathies are thus a secondary consequence of Schwann cell defects (Feldman et al., 2017; Gonçalves et al., 2017).

Why in diabetic neuropathy, small sensory axons are more prone to degeneration compared to motor axons is not yet understood. Possibly, the higher vulnerability might be explained by an accumulation of toxic waste products that are not as efficiently removed or possibly more concentrated than in larger motor axons. Alternatively, larger axons do not rely as much on glial support as smaller axons. This might be corroborated by the finding that Schwann cell-specific loss of the serine threonine kinase LKB1, which regulates cellular metabolism downstream of adenosine monophosphate-activated protein kinase, leads to a preferential degeneration of small-diameter axons as found in diabetic neuropathy.

The recent findings made in the Drosophila system may shed some new light on the question whether metabolic support is sufficient to explain sensory axon degeneration. In flies, small sensory axons are generally found in larger fascicles. In these fascicles, only the marginal axons establish contact with glial cell processes (Kautzmann et al., 2025). Thus, one would expect that the glial contact *per se* is not essential. In contrast, motor axons are intensively engulfed by glial processes and therefore appear to depend on this contact more than sensory neurons. Upon ablation of wrapping glia, however, only sensory neurons degenerate (**Figure 1**). Thus, glia cells either secrete a sensory neuron-specific survival factor, or sensory neurons accumulate toxic products because they lack an efficient secretion system that is installed by motor axons.

These questions are not easily addressable in a mammalian model system. In flies, however, the sophisticated genetic manipulation techniques will allow to conduct cell type-specific loss-of-function or gain of function screens. In the future, for example, it will be possible to screen for gene functions in neurons that either prevent or stop axonal degeneration. Similarly, we can use axonal degeneration models in which the glia is still partially present. Here, we can look for glial functions that allow the prevention of axonal degeneration. Such experiments will not only inform on why neuronal modality matters when reacting to loss of glia, they may also open new avenues towards a more effective therapy of diabetic- and chemotherapy-induced peripheral neuropathies.

*This work was funded by a grant of the Deutsche Forschungsgemeinschaft (DFG) (SFB 1348, B5) to CK.*
